# Biological puzzles solved by using *Streptococcus pneumoniae*: a historical review of the pneumococcal studies that have impacted medicine and shaped molecular bacteriology

**DOI:** 10.1128/jb.00059-24

**Published:** 2024-05-29

**Authors:** N. Luisa Hiller, Carlos J. Orihuela

**Affiliations:** 1 Department of Biological Sciences, Carnegie Mellon University, Pittsburgh, Pennsylvania, USA; 2 Department of Microbiology, Heersink School of Medicine, University of Alabama at Birmingham, Birmingham, Alabama, USA; Geisel School of Medicine at Dartmouth, Hanover, New Hampshire, USA

**Keywords:** *Streptococcus pneumoniae*, discovery, pathogenesis, vaccinology, molecular biology, genetics, model organism

## Abstract

The major human pathogen *Streptococcus pneumoniae* has been the subject of intensive clinical and basic scientific study for over 140 years. In multiple instances, these efforts have resulted in major breakthroughs in our understanding of basic biological principles as well as fundamental tenets of bacterial pathogenesis, immunology, vaccinology, and genetics. Discoveries made with *S. pneumoniae* have led to multiple major public health victories that have saved the lives of millions. Studies on *S. pneumoniae* continue today, where this bacterium is being used to dissect the impact of the host on disease processes, as a powerful cell biology model, and to better understand the consequence of human actions on commensal bacteria at the population level. Herein we review the major findings, i.e., puzzle pieces, made with *S. pneumoniae* and how, over the years, they have come together to shape our understanding of this bacterium’s biology and the practice of medicine and modern molecular biology.

## INTRODUCTION

Sir William Osler described pneumonia as “the captain of the men of death” (1). If so, then *Streptococcus pneumoniae* (the pneumococcus) has long been and remains a stalwart soldier in this legion of death. Most likely first described by Klebs in 1875 (2), it was not until 1881 that both George M. Sternberg and Louis Pasteur independently described the ability of these lancet-shaped, ovoid diplococci to kill their host (3, 4). Pasteur injected saliva from a child who had died of rabies into rabbits, whereas Sternberg did the same with his own saliva. In both instances, the rabbits died, and diplococci were isolated from the bloodstream. Sternberg named the isolated bacteria *Micrococcus pasteuri*, while Pasteur named this new pathogen *microbe septicemique de la salive*. By 1886, it was referred to in publications by Fraenkel as “pneumococcus” due to its frequent isolation from individuals with pneumonia (5). In 1920, it was renamed *Diplococcus pneumoniae* (6). Only in 1974 was this bacterium given the moniker *Streptococcus pneumoniae* due to its growth as short chains when grown in media (7).

**Fig 1:**
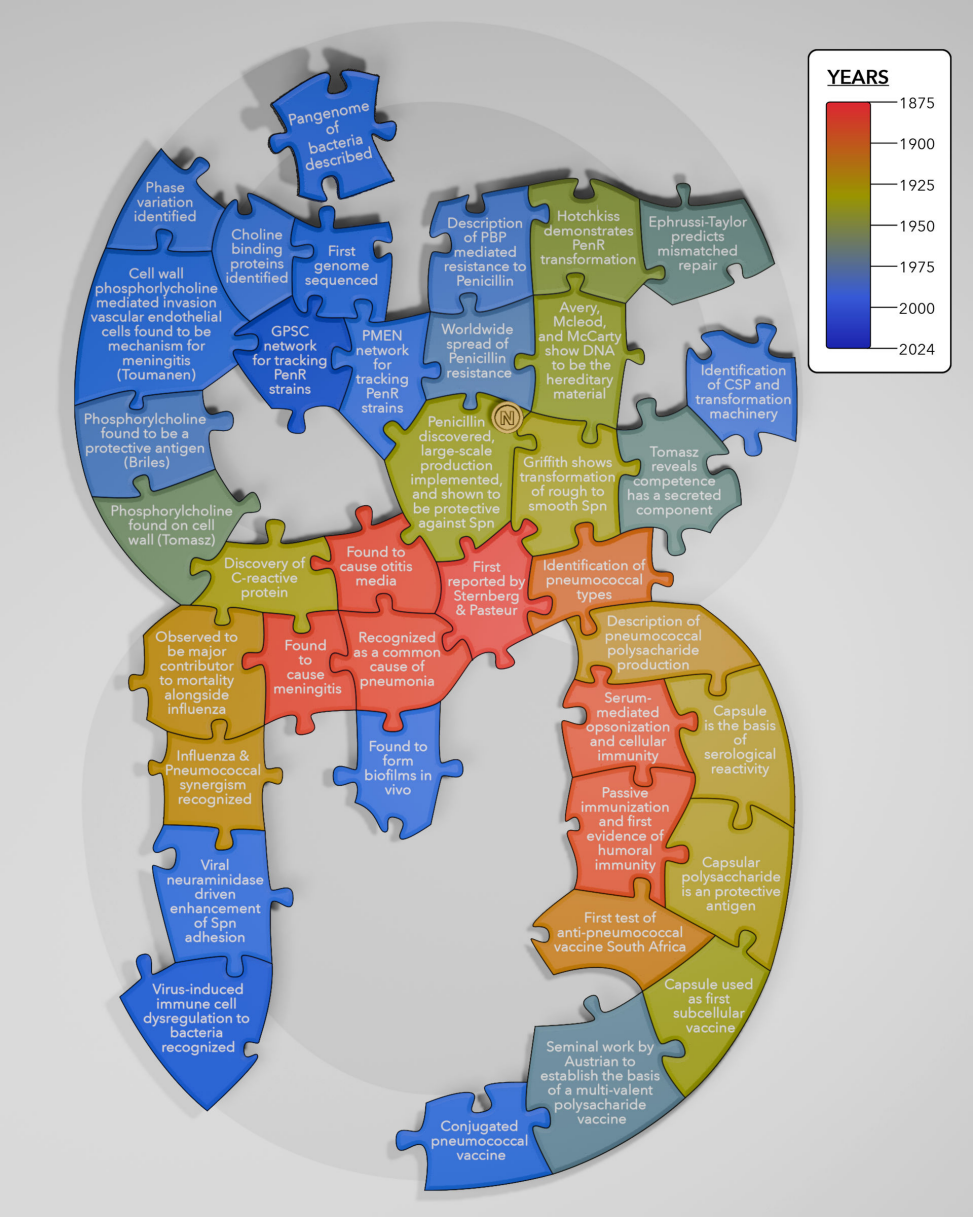
The pneumococcus-shaped puzzle highlights major discoveries associated with this bacterium and captures the following key areas of discovery: identification of the species and its association with pathogenesis (central), its central role in the discovery and validation of polysaccharide vaccines (lower right), discoveries related to the transforming principle and gene transfer (upper right), genome sequencing, strain tracking and pangenome studies (top), viral-bacterial interactions (lower left), and characterization of cell wall components (upper left). Puzzle pieces are color coded according to time of the discovery, with empty areas representing yet undiscovered facets of pneumococcal biology or pathogenesis. Created by Emily Krueger, reproduced with permission.

Soon following its discovery, *S. pneumoniae* was recognized to be a common cause not only of pneumonia but also of otitis media and meningitis (8, 9). Importantly, during the late 1800s and early 1900s, and with a notable spike as a result of the 1918 influenza pandemic, pneumonia was the third leading cause of death overall with *S. pneumoniae* as a primary culprit (10, 11). Accordingly, *S. pneumoniae* was extensively studied, and during this golden era of discovery, it was in many instances the microbe used to first describe and subsequently characterize fundamental biological, clinical, and immunological phenomena. These lines of study ultimately resulted in the development of polysaccharide-based vaccines, which have saved the lives of millions (12–14). Additionally, studies revealed DNA as the unit of inheritance (15) marking the start of modern molecular genetics. Subsequent work with *S. pneumoniae* has pushed the boundaries of our understanding of genetic plasticity, species diversity, and the evolution of bacteria in response to human action. These and other major milestones associated with our understanding pneumococcal pathogenesis are detailed below (Fig. 1).

## SEROTYPING AND POLYSACCHARIDE-BASED VACCINES

In 1891, Klemperer and Klemperer reported that serum from rabbits injected with heat-killed pneumococci or *in vitro* broth culture filtrates contained a factor that when infused into another animal conferred protection against lethal challenge with the same strain but not necessarily other clinical isolates (16). In turn, Metchnikoff and Issaeff separately showed that this generated serum could aggregate the bacteria and enhanced the uptake of pneumococci by phagocytes, respectively (17, 18). These were among the first demonstrations of humoral and cellular immunity. Subsequent work by Neufeld showed that S. *pneumoniae* consisted of distinct “types” to which serum-based immunity could be categorized; i.e., serum to bacteria in one type would not react with bacteria belonging to another type and, when bacteria were mixed with specific antiserum against the same type, would cause agglutination visible to the human eye, and that under a microscope, “quellung” or swelling of the bacterium’s capsule occurred (19). Differential reactivity to antiserum became the basis of pneumococcal serotyping, which remains the most common form of classification for *S. pneumoniae*. It was not until 1917 that Dochez and Avery first determined that the pneumococcus produced copious levels of polysaccharide during infection (20). Subsequently in 1923, Avery and Heidelberger showed that this polysaccharide was the antigenic basis of serological reactivity (21). This discovery revealed that factors other than proteins could be antigens and expanded the potential repertoire of future vaccine formulations. Today, more than 100 distinct serotypes of *S. pneumoniae* have been identified. What is more, the biochemical composition of most capsule types has been defined and is now known to be responsible for the antibody-based differences in reactivity (22–24).

Even before capsule was identified as a protective antigen, efforts were under way to determine whether immunization was a viable means to block pneumococcal disease. In 1911, Sir Almroth Wright tested if whole killed pneumococci protected African gold miners against pneumococcal infection (25). His failure to demonstrate efficacy was likely the result of a low dose of antigen administered to the miners as well as the mixture of pneumococcal serotypes causing disease, versus the single strain used for immunization (26). In 1937, immunization with purified capsular polysaccharide was used to stop an outbreak of pneumonia in a state hospital (27). This was the first demonstration of a subcellular vaccine having efficacy against disease. Others who published findings supporting the efficacy of immunization with *S. pneumoniae* products during this time include Macleod, Heidelberger, and Kaufmann and their respective colleagues (26, 28). In later years, Robert Austrian published seminal work on the topic showing that a polysaccharide vaccine was efficacious for at-risk adults (29, 30). The work was soon extended to include not only capsule vaccine against pneumococcal disease but also capsule vaccine targeting *Haemophilus influenzae* and *Neisseria meningitidis*. Ultimately, this body of work led to the creation and licensing of a purified polysaccharide vaccine containing capsules corresponding to 14 different serotypes, which at the time accounted for 80% of pneumococcal disease in the United States. This was expanded to 23-valent in 1983 (31), and this formulation remains licensed for use in adults today.

Following the introduction and success of the protein conjugated *Haemophilus influenza* type b vaccine developed by Robbins et al. (32, 33), a new seven-valent conjugate vaccine against *S. pneumoniae* was tested in the 1990s. This version was composed of purified capsular polysaccharide conjugated to the diphtheria toxoid CRM197, conferring reactivity as a T cell-dependent antigen and thus protection of children under the age of 2 years. This new pneumococcal vaccine, subsequently licensed in 2000, was found not only to be efficacious against pneumonia and invasive disease caused by the included serotypes (34, 35) but also to prevent colonization of the nasopharynx, thereby blocking transmission and conferring herd immunity to unvaccinated individuals (36, 37). Based upon this success, but also to thwart the escalation of non-vaccine serotypes, which moved into this unoccupied niche (38, 39), two new conjugate vaccines containing 10 and 13 capsule types were introduced in 2010 (40). The newest versions of these vaccines, 15- and 20-valent, were approved for use in the United States in 2022. Notably, in other countries, *S. pneumoniae* conjugate vaccines are designed to address their local distribution of serotypes and thus contain a different set of capsular polysaccharides. The preponderance of data indicates that the pneumococcal conjugate vaccine is a major public health triumph, with rates of disease caused by almost all serotypes included within these vaccines having plummeted since their introduction.

Importantly, the licensing of these newer conjugate vaccine formulations was not dependent on large clinical trials testing efficacy against disease, but instead on demonstration that the new vaccine generated a comparable immune response to the serotypes covered by the older conjugate vaccine, that antibody titers against any new capsule types reached a predetermined titer considered to be protective (41), and that the newly generated antibody demonstrated efficacy in an *in vitro* opsonophagocytosis killing assay (42). This change in licensing requirement and its acceptance by the World Health Organization (WHO) were made possible due to the development of reliable assays for measuring antibody levels and their function (43, 44). In the wake of the success of these vaccines, the approaches used for the pneumococcal vaccines have become the model for developing polysaccharide-based vaccines against many other pathogens.

## PENICILLIN AND THE TREATMENT OF PNEUMOCOCCUS

In 1926, Felton and Bailey were able to purify capsular polysaccharide and show that this was the subcellular fraction in heat-killed bacteria responsible for conferring immunity (45). With the understanding that serum containing antibody against capsule was protective, serotherapy, the use of animal-generated antiserum, was in use to treat *S. pneumoniae* infections by 1913. This treatment reduced mortality from 25.0% to 7.5% (46). Serum therapy relied on first obtaining sputum, followed by serotyping to select the matching antiserum. Thus, this process is perhaps the first example of what is now referred to as personalized medicine (47). Serum therapy was subsequently abandoned due to efficacy of a powerful new form of treatment: antimicrobials (28). In 1931, sulfapyridine was introduced and even used in 1942 to treat Winston Churchill’s pneumonia (48). However, the use of sulfapyridine was soon replaced with penicillin. Penicillin was discovered in 1929, when Fleming characterized a mold contaminant that induced lysis of staphylococcus colonies (49). Twelve years after Fleming’s discovery, a team of British scientists led by Florey and Chain published a groundbreaking and comprehensive study on this cell wall-acting antibiotic, which set the stage for penicillin to revolutionize human medicine. The study described methods for purification of penicillin, its bacterial targets, its efficacy in killing bacteria on cells, animal models, and five patients with pneumonia (50). Fleming and Florey and Chain were awarded the 1945 Nobel Prize in Physiology and Medicine for these contributions. The potential of this new medicine for treatment of wounded World War II soldiers was immediately recognized, with Florey leading a trial in North African military hospitals in 1942 (51). In 1943, penicillin was administered to 500 patients for the treatment of streptococcal infections in the United States and was found to be highly effective against pneumococcal pneumonia (28, 52). Subsequent widespread implementation of penicillin resulted in a 40%–50% decrease in *S. pneumoniae*-associated mortality among those 12 years of age to the elderly (28, 53). In fact, vaccine development came to a complete halt because of the effectiveness of penicillin. However, in 1964, Austrian and Gold reported that penicillin had no effect on the outcome of bacteremic pneumococcal pneumonia over the first 5 days of infection (54). This meant that the arsenal against the “captain of the men of death” needed to be expanded.

While penicillin analogs today remain a common treatment for pneumococcal infections, the second half of the 20th century was marked by global waves of penicillin resistant (PenR) *S. pneumoniae*. The first clinical cases of resistance for *S. pneumoniae* were seen in Boston in 1965, albeit they were not recognized as such (55). Subsequently, PenR *S. pneumoniae* was reported in Australia in 1967, and by 1974, resistant strains were reported worldwide, with high incidence in South Africa, Hungary, and Spain (55–58). Levels of resistance increased dramatically over the next decades. For example, in a Spanish hospital, the incidence of PenR *S. pneumoniae* went from 4% in 1979 to 40% in 1990 (57). While rates of PenR invasive infections in the United States remain highly variable across time and geographic regions (59), it is clear that the conjugate vaccine has impacted antimicrobial resistance rates by reducing the prevalence of *S. pneumoniae* serotypes whose genome encodes antibiotic resistance markers. In this manner, the inclusion of select serotypes in the vaccine is also a means to block the spread of antibiotic-resistant strains. Highlighting the potential risks associated with the spread of this pathogen, today, penicillin non-susceptible pneumococci are categorized as priority 3 by the WHO and drug-resistant *S. pneumoniae* as a serious threat by the Centers for Disease Control and Prevention (60, 61).

In contrast to most PenR bacteria, the pneumococcus does not produce a β-lactamase that destroys the antimicrobial. Instead, as described in the 1980s in clinical isolates of pneumococci and *Neisseria gonorrhoeae*, PenR in the pneumococcus arises from modifications in wall transpeptidases [the penicillin binding proteins (PBPs)] that decrease their affinity to this antibiotic (62–64). This alternative mechanism of resistance is common in multiple species of enterococci, *Neisseria*, and other streptococci, as well as *Staphylococcus aureus* (65, 66). Sequence comparisons and genetic studies comparing PenR and sensitive strains in the 1980s and 1990s provided global and fine-tuned understanding of the structure-function relationships between PBPs and penicillin and revealed recombination of the DNA encoding PBPs between *S. pneumoniae* and related *Viridans* streptococci that resulted in this trait (57, 67–74). These studies lead to the prevailing model that intra- and interspecies recombination events are a prominent mechanism for development of PenR in pneumococci (70, 75). This model continues to be widely accepted and is further supported by *in vitro* evolution studies (76). Together, the high fitness costs of *de novo* generation of PenR and the high rates of gene transfer in *S. pneumoniae* have led to enormous diversity in the array of PBPs found in PenR pneumococci.

Given that the capsule gene locus is frequently a site for exchange by genetic recombination, genetic lineages of *S. pneumoniae* often encompass multiple different serotypes. Along such lines, the need to track antibiotic resistance for *S. pneumoniae* beyond their serotype was a major impetus for developing tools to categorize the genetic distribution of specific genetic lineages. One of the first comprehensive ways to determine differences was via pulse-field electrophoresis of genomic DNA (77). Subsequently, and as PCR and DNA sequencing became readily available, multilocus sequence typing became the dominant methodology (78). In 1997, the Pneumococcal Molecular Epidemiology Network was established to better characterize and standardize the identification of antimicrobial-resistant pneumococcal clones (79). More recently, with the emergence of what is perhaps the most extensive collection of sequenced genomes among clinical bacterial isolates as well as the widespread feasibility to sequence new isolates, the Global Pneumococcal Sequencing (GPS) Project (GPS Database) has established a worldwide surveillance network to collect genomic sequence and epidemiological data on strains, including antimicrobial susceptibility (80) (https://www.pneumogen.net/gps/index.html). With over 21,000 whole genomes of *S. pneumoniae* now publicly available, our understanding of pneumococcal variability and the extent of horizontal gene transfer can be studied at an unprecedented level of resolution. All in all, the discovery and industrial-scale implementation of antimicrobials to treat bacterial infection were revolutionary in human medicine. The subsequent spread of PenR *S. pneumoniae* highlights the stark ability of this pathogen to change population structure in response to human-based intervention. Finally, the human need to track the epidemiology of resistant strains has in turn served as a window to understand intraspecies genomic variability and plasticity (81).

## ANTIBIOTIC TOLERANCE, HETERORESISTANCE, AND AUTOLYSINS

Not all bacteria that survive exposure to antibiotics are resistant, i.e., able to grow in the presence of antimicrobials. Instead, some are tolerant, capable of resuming growth after removal of the antimicrobial. In 1970, Tomasz et al. observed that suppression of the autolytic enzyme LytA led to absence of lysis and killing by cell wall active antibiotics (82). His group went further to describe that escaping the killing activity of antibiotics could be by virtue of genetic mutation (irreversible) or phenotypic growth conditions (reversible), that non-growing bacteria do not necessarily die, and that the rate of growth was directly proportional to the rate of death (83, 84). These findings underpin the field of antibiotic tolerance.

The pneumococcus also exhibits heteroresistance (85). In this case, subpopulations within a monoclonal culture exhibit the ability to grow at antibiotic concentrations above the minimal inhibitory concentration. This resistance is reversible, consistent with a non-heritable response. Yet, the property of heteroresistance is also strain specific, suggesting heteroresistance is influenced by factors encoded in the genome. Along such lines, this property has been associated with the number of altered PBPs, specific low-affinity alleles of *pbp2x*, and induction of phosphate ABC transporter genes (85, 86). It is tempting to speculate that heteroresistance results from hedge betting behaviors associated with survival in the presence of penicillin. At this time, the genetic determinants or molecular networks that determine the probability of heteroresistance within a population and their clinical consequences remain poorly understood. While the spread of PenR is an undeniable threat to global human health, the clinical consequences of tolerance and heteroresistance remain less clear, yet they likely contribute to relapsing infections and may serve as an intermediate step toward the evolution of resistance.

## THE TRANSFORMING PRINCIPLE AND GENETIC PLASTICITY OF *S. PNEUMONIAE*

Transformation was first discovered in the pneumococcus, and today this bacterium still stands as a paradigm for genomic plasticity. Whereas the clinical importance of horizontal gene transfer is highlighted in the evolution of PenR, our understanding of it started in 1928 when Griffith showed that co-injection of live, attenuated, rough (unencapsulated) pneumococci, formerly type II, with heat-killed encapsulated virulent pneumococci belonging to type III, resulted in death of challenged mice and that only encapsulated bacteria carrying type III capsule were recovered. Thus, rough pneumococci were able to incorporate an element, a “transforming principle,” from the dead bacteria that allowed them to acquire a distinct capsule type and the virulent phenotype (87). Subsequently, in 1944, the nature of this transforming principle was determined in experiments by Avery et al. Using pneumococcal extracts which had carbohydrates and lipids removed, they found that it was the samples treated with DNAse and not proteases that lost their capacity to transform rough pneumococci (15). In 1951, these results were validated by Hotchkiss, who demonstrated that DNA was also responsible for the transformation of PenR (in a capsule-independent manner) (88). The discovery and characterization of DNA as the transforming principle are considered by most to be the start of molecular genetics.

The transfer of DNA across *S. pneumoniae* strains set the stage for subsequent discoveries. Ephrussi-Taylor and Gray puzzled over the different efficiencies in transformation among strains and in 1966 proposed the existence of the cellular mismatch repair system, which they referred to as the “destruction choice” due to strain-dependent bias in the frequency of incorporated DNA sequences in the recombination site (89). Similarly, and also in the 1960s, Tomasz and colleagues established that a secreted protein was required for the ability of *S. pneumoniae* to take up DNA and undergo homologous recombination, providing the first evidence for the mechanism underlying transformation and the first example of a pheromone establishing communication between bacteria (90–92). Two decades later, the nature and processing of the competence-inducing peptide were established by Morrison et al. and Havarstein et al. (93, 94). This secreted peptide, a quorum signal, directs population-level behavior when in sufficient concentration, triggering the assembly of a channel for DNA capture and import, as well as transcriptional changes in over 5% of the genome (95, 96). Notably, and in mixed strain populations, activation of competence can activate a second quorum signal that triggers bacteriocin release and the death of neighboring subpopulations (fratricide), which serve as a source of DNA for genetic exchange (97–99).

It is now clear that *S. pneumoniae* population-level behaviors rely on a multitude of cell-cell communication peptides. These secreted peptides function not only to regulate DNA uptake and competition but also for physiological processes, including nutritional responses, biofilm development, and capsule levels (100–109). Accordingly, many of these peptides are required for colonization and virulence (110). Thus, throughout the 20th century, studies on the transfer of genetic material in the pneumococcus were pivotal for the identification of DNA as the hereditary molecule, shed light on the molecular mechanisms underlying capsule switching (which remained relevant in the context of conjugate vaccine design), and contributed to our understanding of penicillin resistance, cellular mismatch systems, as well as components of quorum sensing and fratricide in bacterial communities.

## STUDIES ON CELL MORPHOLOGY

The diversity of bacterial cell shapes in nature results from varied mechanisms of cell growth and division; the pneumococcus being a prototype for ovoid bacteria (111). This shape is formed by two modes of peptidoglycan synthesis: peripheral and septal. These processes are carried out by the elongasome and divisome, respectively (111). Recent studies in *S. pneumoniae* have shown that the movement of the septal peptidoglycan synthase occurs along a single track at the midcell, propelled by cell wall synthesis (112). Additionally, studies using high-resolution fluorescence microscopy have uncovered the spatial and temporal coordination of both types of peptidoglycan synthesis (113, 114). Highlighting its role as a model organism, studies are uncovering similar patterns of cell wall in other Gram-positive species (115, 116). Finally, while some aspects of *S. pneumoniae* cell division and growth are widespread, this bacterium also displays atypical features regarding the peptidoglycan, such as minimal turnover, differences in metabolic control of the synthesis precursor pathway, and specialization of its synthesis proteins. Current models suggest ovoid shaped bacteria evolved from a rod-shaped ancestor by gene reduction (117).

## THE PNEUMOCOCCAL PANGENOME

In the 1670s, van Leeuwenhoek focused his microscope and observed the bacterial world. Similarly, the development of high-throughput DNA sequencing has revealed another layer of the unseen, the bacterial genome. The first gap-free annotated *S. pneumoniae* genomes were released in 2001, placing them among the first 30 complete genomes released for a human pathogen (118, 119). By 2005, two groups working in parallel concluded that the pangenome of a species extended well beyond the genes of a single strain, leading to the concept of the core genome, accessory genome, and pangenome (also referred to as supragenome in earlier studies) (120–122). The pneumococcal pangenome was one of the early ones to be described (123, 124). Today, as one of the bacterial species with the greatest number of sequenced genomes, it serves as a valuable model for studies on the characterization and evolution of pangenomes.

The pangenome of *S. pneumoniae* is both large and highly diverse (125). It encompassed the entire set of genes in a population, and the genes are classified based on their distributions, where core genes are encoded in all strains and accessory genes are encoded in a subset of strains. In an individual strain, approximately one-fifth of the genome consists of accessory genes, and together with single nucleotide polymorphisms (SNPs), these underlie much of strain diversity. The percentage of core and accessory genes in the pangenome is database dependent. Early estimates suggested 50% of the genes in the pangenome were core. Yet, in some data sets, the pangenome is over 10,000 genes, making the numbers of core genes as low as 5% (123, 124, 126). Diversity in the pangenome is likely restricted to gene variants that allow for colonization of the human upper respiratory tract, as this niche is the primary reservoir for *S. pneumoniae* and the source for its transmission. Our personal observation is that often regions of the genome encode functionally related genes that are not homologs, for instance, restriction enzymes or bacteriocins, highlighting a high degree of interchangeability and specialization among accessory genes (97, 127). Notably, analyses of genetic lineages generally do not reveal a robust correlation between gene content and the ability to successfully colonize a specific niche in the human host. Exceptions exist, such as the classic non-typable strains which lack capsule, i.e., non-encapsulated *S. pneumoniae*: these form a distinct phyletic group and are associated with eye infections (128–131). The plasticity of the *S. pneumoniae* genome extends beyond gene diversity and also includes duplication events, as illustrated by a recent study where suppressor mutations have arisen via dosage effects incurred from chromosome duplications (132). Studies of the pneumococcal pangenome have not only shed light on the diversity and plasticity of this species but have also served as an important and mathematical framework for the study of evolution. Mathematical models suggest that the frequencies of accessory genes are shaped by negative frequency dependency, where rare genes are selected for until they become common, at which point they incur a cost, for example, as result of antibody recognition due to prior colonization by another strain carrying the same gene product (133). This suggests a very robust distribution of genes within the pangenome with an equilibrium population composition. Modeling this state provides a framework within which to assess the threat of emerging lineages and predict the impact of interventions, i.e., antimicrobial and vaccine-based, on pneumococcal populations (133). Overall, the plasticity of single strains, incurred by horizontal gene transfer and supported by a diverse pangenome, may allow strains to adapt to host niches and therapeutics. However, the structure of the pangenome, including the frequency at which individual genes are observed within the population, may be constrained. Thus, the *S. pneumoniae* population is shaped by factors that promote both flexibility and constraint.

Classically, a molecule that when blocked (by gene deletion or inhibition of function) leads to a decrease in virulence or virulence-associated phenotypes is termed a virulence factor. However, it is noteworthy that an estimated 80% of *S. pneumoniae* virulence determinants, including the genes encoding capsular polysaccharide production and the pore-forming toxin pneumolysin, have orthologs encoded in closely related commensal species or are part of the core genome and found in non-virulent versions of *S. pneumoniae*, suggesting that their presence alone does not confer pathogenicity (123). Along such lines, transposon-based mutagenesis studies performed in the 1990s and 2000s supplemented by comparative genomic studies and targeted mutagenesis of genes encoded on regions of diversity or pathogenicity islands further support the idea that disease can be potentiated by different bacterial factors and their interplay with diverse host factors (134–136).

Differences between infected individuals also determine what a virulence determinant is. A recent genome-wide association study on a large number of samples of patients and pathogens of pneumococcal meningitis revealed that the genetic diversity of the host may explain almost 50% of the variation in disease severity, shedding light on the extent to which host and bacterial components contribute to invasive disease (137). Moreover, there is also evidence that the fitness landscape within the host drives the genomic composition of the pneumococcus. This is elegantly illustrated by a study of strains isolated from patients with sickle cell disease over a 20-year period (these patients display a 600-fold increase in pneumococcal mortality) (138). The analyses revealed a set of virulence determinants that is distinct from the set in the general population and likely selected for by the high-iron environment, the chronic activated endothelium, and the long-term penicillin pressure in individuals with sickle cell disease. In this context, virulence can be conceptualized as an emergent property driven by both host and pathogen determinants, where the molecular determinants of the pathogen can be shaped by host conditions.

In summation, studies focused on determining the basis of antimicrobial resistance made it clear that *S. pneumoniae* should not be sorted based on serotype alone. Pangenome studies emphasized this perspective by revealing unexpected diversity across genetic lineages and even within strains belonging to the same lineage. Further, characterization of the pangenome has offered a conceptual framework to consider *S. pneumoniae* as a dynamic community that exchanges genetic material across strains and related species, resulting in genomic variability and the ability to overcome new and unpredicted selective pressures imposed by the host. Not surprisingly, it is noteworthy that *S. pneumoniae* is a prototype for the development of CRISPR-based gene expression knockdown systems to assess the contribution of gene products to bacterial fitness without the limitation imposed by gene deletion-mediated bottlenecks (139, 140).

## BIOFILM FORMATION DURING COLONIZATION AND GROWTH WITHIN TISSUES

*In vivo* studies with *S. pneumoniae* have affirmed that the biofilm state is of major consequence during nasopharyngeal colonization, not necessarily associated with a disease state, and a major contributor to the antimicrobial recalcitrance seen during otitis media. Host factor-triggered dispersal of *S. pneumoniae* in a biofilm is also a mechanism as to how pneumococci transition from asymptomatic colonizers to potentially deadly opportunistic pathogens. The concept and term “biofilm” was coined and crystalized by J. Willian “Bill” Costerton during his studies of environmental bacteria (141, 142). Work in the 1980s introduced the importance of this mode of bacterial growth in chronic infections including cystic fibrosis and infected internal prosthetic devices (143). In a series of four studies, Ehrlich and colleagues demonstrated the role of biofilms in pneumococcal disease when they demonstrated that bacterial biofilms contribute to the pathogenesis of chronic otitis media (144–147). Over the next decade, biofilms were characterized on abiotic surfaces, on cultured cells, and *in vivo* (148–151). Further, the genomic determinants of biofilms were explored, demonstrating, for example, that the capsule is highly inhibitory to biofilm formation (152). For *S. pneumoniae*, the phenotypic implications of growing as biofilms include heightened transformation efficiency, increased tolerance to antimicrobial agents, resistance to desiccation, facilitation of survival on inanimate objects (fomites), and a diminished capacity for invasiveness and immunoreactivity (153–158). Today, biofilm formation is thought to occur as a metabolic response to the bacterium’s environment and to be a key part of the complex interplay between bacterial and host factors. Correspondingly, changes in the host, such as viral infections, microbiota switches, or inflammatory responses, are thought to trigger biofilm dispersal (157). Bacteria dispersed from biofilms exhibit gene expression signatures that differ from both planktonic and biofilm modes of growth and display an enhanced ability to cause infection in murine models of disease (159). Interestingly, there is evidence that *S. pneumoniae* that are attached to tissue are behaving like biofilms, perhaps due to depletion of nutrients at the level of the infected microenvironment (160). Importantly, capsule production is one of the virulence determinants most strongly affected by carbon availability, and since its presence inhibits attachment and biofilm formation, there is a direct link between metabolism, physiology, and virulence that is increasingly reinforced by emerging metabolism-related studies.

## VIRAL BACTERIAL SYNERGY

Respiratory viruses have long been recognized to potentiate bacterial infection with the most severe forms of pneumococcal pneumonia superimposed or closely following a preceding viral infection (161–164). The latter is especially the case for influenza A virus (165), and *S. pneumoniae* superinfection contributed substantially to the mortality associated with the 1918–1919 influenza pandemic (10, 11). As a result and over the years, scores of studies having explored the molecular mechanisms underlying this synergism, and in many ways, influenza and *S. pneumoniae* have together served as the prototypes to understand how respiratory viruses exacerbate airway bacterial disease. For example, influenza A virus is known to bind to the surface of *S. pneumoniae* and other bacteria, enhancing their capacity to adhere to host cells (166). Viral-induced inflammation and influenza neuraminidase activity frees nutrients and exposes receptors for aspirated bacteria to co-opt (167, 168). Influenza disrupts the function of the mucociliary escalator (169) and also causes macrophages to lose efficiency in the uptake of bacteria via scavenger receptors such as MARCO (170). Influenza-induced ion channel dysregulation also causes an increase in the pH of mucosal secretions that reduces the activity of antimicrobial components (171). Pneumococcal gene expression is altered following interactions with influenza and influenza-infected cells. This exposure triggers dispersal from biofilms as well as altered expression of virulence genes including increased expression of pneumococcal surface protein A (PspA), which mediates pneumococcal attachment to dying host cells (157, 172, 173). The latter is important for transmission, as bacteria attached to host cells are shed during colonization, and this enhances their resistance to desiccation (174, 175). Further, influenza has been shown to prime mucosal epithelial cells for pneumolysin-mediated necroptosis and NLRP3-inflammasome-driven pyroptosis during co-infection (158, 176), enhancing disease severity in the airway by altering localization of the bacteria and further releasing factors *S. pneumoniae* uses for its metabolic benefit (177). Notably, and alongside other work showing that other viruses such as respiratory syncytial virus, rhinovirus, and metapneumovirus potentiate pneumococcal disease, recent work suggests there is also a lethal synergy between severe acute respiratory syndrome coronavirus 2 (SARS-CoV-2) and *S. pneumoniae* (178–181). Moreover, while superinfections are relatively rare, they drive a meaningful fraction of SARS-CoV-2-associated mortality (182). One explanation is that *S. pneumoniae* colonization dampens the antiviral response, in turn negatively impacting the generation of antibodies against SARS-Cov-2 (183). Thus, pneumococcus is again at the forefront of attempts to understand the basis of severe respiratory disease.

## COLONIZATION FACTORS, PHASE VARIATION, AND INADVERTENT DISEASE

The study of *S. pneumonia* has also contributed to our general and molecular understanding of how asymptomatic colonizers of the upper airway adapt to the host as disease develops. Sequencing of the *S. pneumoniae* genome revealed that a major portion of open reading frames was dedicated to acquisition and utilization of diverse carbon sources (119), a feature that most likely reflects the paucity of glucose in the human nasopharynx (184). Importantly, the purpose of *S. pneumoniae* virulence factors is to promote colonization and transmission to the next host (185, 186). The fact that these determinants are also responsible for disease is incidental to their role in colonization and transmission. With this mindset, investigators have performed genetic screens to identify the factors that are important for transmission and not surprisingly have identified several non-canonical virulence determinants. These included the *dlt* locus, a determinant of antimicrobial peptide resistance, which enhances pneumococcal shedding by adding d-alanine onto lipoteichoic acid and thereby increasing toll-like receptor recognition and localized inflammation, which promotes mucous secretion and with it bacterial expulsion (187, 188). Likewise, genes involved in DNA repair pathways were found to be required for desiccation resistance (189). Genes involved in fatty acid metabolism, oligopeptide transport, biosynthesis of amino acids, and iron transport have also been identified as being critical. Altogether, these studies suggest that resilience and metabolic aspects are key for the transmission process and that bacteria must be able to accommodate the harsh transitionary environment on a fomite as well as the naïve host from a metabolic perspective (190, 191).

In 1994, Weiser et al. described pneumococcal phase variation for the first time. In brief, the bacterium was found to spontaneously, and at low incidence, change between a transparent and opaque colony phenotype when grown as single colonies on clear agar plates and viewed with oblique light (192). Since then, phase variants have been shown to be different with regard to their expression of multiple surface proteins, levels of capsular polysaccharide and cell wall teichoic acid (or C-polysaccharide), and hydrogen peroxide production, which is a by-product of pyruvate oxidase activity, among other aspects (193–195). Further work led to the understanding that the transparent phenotype, which is more adhesive and carried less capsule, is the predominant phenotype present in the nasopharynx during colonization. In contrast, the opaque phenotype is the version found in the bloodstream (196). Subsequent work showed the opaque phase variants are better protected from immune cells that are present under inflammatory conditions such as otitis media and following an exacerbating event, such as during viral co-infection (197). Importantly, the mechanism for phase variation has been identified as a reversible shift in the methylation pattern caused by DNA inversions in three homologous DNA methyltransferases that are part of a restriction modification system (198, 199). These modifications impact the transcription level of genes across the genome and illustrate how pneumococcal epigenetics serve as an additional layer of single-cell diversity.

In summary, and despite being a major cause of human mortality, *S. pneumoniae* should be considered in the context of its adaptation to its obligate human host, as a pathobiont—typically asymptomatic colonizer that can cause disease under proper circumstances—which must persist in the upper respiratory tract long enough for transmission to a new host via aerosols and fomites. In turn, we must also consider the epigenomic, transcriptional, and post-transcriptional adaptation that occur once the pneumococcus disseminates away from the nasopharynx to other tissues, causing opportunistic disease (200). The nature and regulation of these virulence factors are most likely selected by fitness advantages associated with robust chronic colonization and greater transmission rates, and not for tissue dissemination or pathogenesis.

## CHOLINE-BINDING PROTEINS AND AN INTRACELLULAR ROLE OF THE PNEUMOCOCCUS

C-reactive protein (CRP) was discovered in 1930 in sera of patients with acute pneumococcal pneumonia and was so named as it bound to the C-polysaccharide, i.e., wall teichoic acid, a component of *S. pneumoniae* cell wall. CRP is now known to be produced by the liver in response to interleukin-6, and the binding of CRP to its ligand, phosphorylcholine residues on wall teichoic and lipoteichoic acid, serves to activate the complement system and opsonize the bacterium (201). In 1967, Tomasz first showed that choline was incorporated on the pneumococcal cell wall (202). Clark and Weiser extended the impact of this finding to show that all respiratory pathogens harbor phosphorylcholine somewhere on their surfaces, not only to bind CRP but also to adhere to the platelet-activating factor receptor on the pulmonary epithelium (203).

Subsequent work by multiple groups, spearheaded by work done on the bacterium’s autolysin LytA and PspA, identified a family of proteins known as choline-binding proteins (204–206). These molecules play critical roles in pneumococcal cell wall remodeling, autolysis, immune evasion, host cell adhesion, and the invasion of host cells and, for this reason, have been considered to be potential vaccine antigens (207). For the most severe forms of disease, *S. pneumoniae* binds and translocates across vascular endothelial cells. Crossing the blood-brain barrier results in meningitis, and crossing into the bloodstream provides a doorway to other organ systems (208–210). Key virulence determinants for pneumococcal binding and translocation across vascular endothelial cells include the aforementioned phosphorylcholine residues present on wall teichoic and lipoteichoic acid, which bind to platelet-activating factor receptor on host cells (210, 211); choline-binding protein A (alternatively PspC), which binds to host laminin receptor and polymeric immunoglobulin receptor (212, 213); and the pneumococcal pilus, when present, which binds to platelet endothelial cell adhesion molecule 1 (214, 215). Critically, these interactions also take place with mucosal epithelial cells and, as such, are important for colonization. Notably, capsule, which is a requisite for pneumococcal survival in the bloodstream, is generally inhibitory of host cell invasion (210). Along such lines, autolysin-mediated capsule shedding was recently discovered as a way for the pneumococcus not only to evade killing by host cationic antimicrobial peptides but also to facilitate its adhesion to lung cells (216). Yet, still, the production of capsular polysaccharide by *S. pneumoniae* and other pathogens has also been shown to be important for translocation across vascular endothelial cells and to delay bacterial killing that results from the maturation of endolysosomes (217). Thus, work with S. *pneumoniae* has shown that the role of capsular polysaccharide on bacterial pathogenesis is multifaceted, its benefits context dependent, and surprisingly includes intracellular survival.

While *S. pneumoniae* is thought of as an extracellular pathogen and paracellular translocation of tissues is known to occur, a transitionary intracellular role is also a key aspect of its pathogenesis (218, 219). Within vascular endothelial cells, *S. pneumoniae* spp. exist within clathrin-coated endosomes which evade degradation by acidic lysosomes in a pneumolysin-dependent manner (220, 221). Current work suggests that the pneumococcus is co-opting aspects of LC3-mediated endocytosis to enter the cell and persist (222). Notably, work with *S. pneumoniae* led to an improved understanding of how bacteria translocate across the blood-brain barrier and even as to why other respiratory tract pathogens are neurotropic. Along such lines, *S. pneumoniae*, *Haemophilus influenzae*, and *Neisseria meningitidis* were all found to bind to platelet-activating factor receptor via phosphorylcholine residues and to laminin receptor (211, 212, 223, 224), albeit adhesion to the latter is through distinct proteins. These interactions initiate the uptake and translocation of bacteria across vascular endothelial cells including the blood-brain barrier (208, 210). Notably, while the majority of these bacteria are killed as a result of this process, some survive and are shuttled to the parenchyma of the organ or released into the central nervous system (210, 217). Intracellular growth within immune cells may also serve as a reservoir for *S. pneumoniae* during invasive disease. Oggioni and colleagues suggest that intracellular survival in CD169^+^ splenic macrophages drive pneumococcal bacteremia following an initial clearance event (37). Intracellular pneumococci have also been implicated in the death of cardiomyocytes and heart failure during severe infections (225). Thus, a better understanding of the often-overlooked intracellular aspect of disease is warranted.

## CHARACTERIZATION OF CHOLINE-BINDING PROTEINS AND THE POTENTIAL FOR A PROTEIN-BASED VACCINE CANDIDATE

Until the 1980s, when Briles et al. showed that antibody against phosphorylcholine was protective against *S. pneumoniae*, capsule was generally thought as the only truly protective antigen (226). In 1984, McDaniel, Briles, and colleagues (227) identified a monoclonal antibody against a protease-sensitive pneumococcal antigen that was protective against challenge (16). The knowledge of this molecule, subsequently called pneumococcal surface protein A (18) and shown to be a choline-binding protein, along with the earlier newfound ability to purify the bacterium’s previously discovered pore-forming toxin pneumolysin (228), opened the possibility of creating a protein-based vaccine against the pneumococcus. Subsequent studies, aided in their identification of targets by newly available access to the bacterial genome, and methodologies to screen transposon mutant libraries ultimately resulted in the identification and characterization of virulence associated factors including additional choline-binding proteins, pathogenicity islands, metal acquisition factors, pili, a serine-rich repeat proteins, and other virulence determinants (134, 135, 205, 229–232). Many of these proteins demonstrated protection as antigens in preclinical animal models of pneumonia and sepsis, particularly when used in combination (233–236). However, enthusiasm for a protein-based vaccines ultimately became tempered by the discovery that considerable variation existed for many of these proteins, and individual versions were not always cross-protective (237). Moreover, evidence emerged that many of these proteins were not uniformly present in all pneumococci (238) . What is more, transcriptomic analyses of pneumococcal gene expression *in vivo* showed considerable variability in gene expression across distinct anatomical sites (160, 239), indicating that the targeted antigen may not be present during some facets of disease. This enhanced understanding of the complexity of any protein-based vaccine occurred in the backdrop of simultaneous continued success of the conjugate vaccine against *S. pneumoniae*, and, in turn, its steady expansion to include the majority of virulent serotypes, further reduced industry support for a protein-based vaccine. Nonetheless, this work resulted in considerable advancement of our understanding of the molecular mechanisms underlying *S. pneumoniae* pathogenesis and how the bacterium interacts with host cells and evades the host defense. Work on protein-based antigens continues today, and most of the effort is focused on identification of antigen(s) to be administered alongside the current conjugate vaccine and thereby provides protection against pneumococci whose serotypes are not included in the current conjugate vaccine formulation. There are also studies on the use of synthetic CPS serotypes coupled to immunogenic proteins (240), as well as the possibility of using pneumococcal extracellular vesicles, as these simultaneously display an array of proteins (241). Potential antigens include well-characterized and established molecules such as PspA and pneumolysin, as well as recombinant hybrid proteins that combine multiple antigens (242).

## SUMMARY

For over a century, studies focused on pneumococcus have sought to reduce this pathogen’s morbidity and mortality. Yet the impact of this work has extended well beyond pneumococcus as associated findings have yielded major breakthroughs in our understanding of genetics, immunology, antibiotic resistance, and evolution among other areas. The ability to induce competence in the laboratory dramatically facilitated the genetic manipulation of *S. pneumoniae*, thereby transforming it into a model organism for multiple areas of study. Current vaccines against *S. pneumoniae* have had remarkable success in reducing the burden of disease and should be considered major public health victories. Yet, despite this, pneumococcus remains a major human pathogen, highlighting the need to improve vaccines and pursue novel antimicrobial targets. Ongoing studies incorporate molecular mechanisms, account for the pneumococcal mode of growth, and consider the impact of the host in the process of pathogenesis. This global perspective on pneumococcal virulence takes into consideration the contribution of bacterial and host nutritional status, immune responses, and surrounding microbes to the pathogenic state. The hope is that these studies will not only address the existing gaps in protection but also continue to uncover fundamental biological tenets.
